# Structural Insight into the Recognition of r(UAG) by Musashi-1 RBD2, and Construction of a Model of Musashi-1 RBD1-2 Bound to the Minimum Target RNA

**DOI:** 10.3390/molecules22071207

**Published:** 2017-07-19

**Authors:** Ryo Iwaoka, Takashi Nagata, Kengo Tsuda, Takao Imai, Hideyuki Okano, Naohiro Kobayashi, Masato Katahira

**Affiliations:** 1 Institute of Advanced Energy, Kyoto University, Gokasho, Uji, Kyoto 611-0011, Japan; iwaoka.ryo.g78@kyoto-u.jp; 2 Graduate School of Energy Science, Kyoto University, Gokasho, Uji, Kyoto 611-0011, Japan; 3 RIKEN Center for Life Science Technologies, 1-7-22 Suehirocho, Tsurumi-ku, Yokohama, Kanagawa 230-0045, Japan; kengo.tsuda@riken.jp; 4 Department of Physiology, Keio University School of Medicine, Shinanomachi, Shinjuku-ku, Tokyo 160-8582, Japan; imait@a8.keio.jp (T.I.); hidokano@a2.keio.jp (H.O.); 5 Department of Chemistry, Keio University School of Medicine, 4-1-1 Hiyoshi, Kohoku-ku, Yokohama, Kanagawa 223-8521, Japan; 6 Institute for Protein Research, Osaka University, 3-2 Yamadaoka, Suita, Osaka 565-0871, Japan; naohiro@protein.osaka-u.ac.jp

**Keywords:** RNA-binding protein, Msi1, solution structure determination, protein-RNA complex

## Abstract

Musashi-1 (Msi1) controls the maintenance of stem cells and tumorigenesis through binding to its target mRNAs and subsequent translational regulation. Msi1 has two RNA-binding domains (RBDs), RBD1 and RBD2, which recognize r(GUAG) and r(UAG), respectively. These minimal recognition sequences are connected by variable linkers in the Msi1 target mRNAs, however, the molecular mechanism by which Msi1 recognizes its targets is not yet understood. We previously determined the solution structure of the Msi1 RBD1:r(GUAGU) complex. Here, we determined the first structure of the RBD2:r(GUAGU) complex. The structure revealed that the central trinucleotide, r(UAG), is specifically recognized by the intermolecular hydrogen-bonding and aromatic stacking interactions. Importantly, the C-terminal region, which is disordered in the free form, took a certain conformation, resembling a helix. The observation of chemical shift perturbation and intermolecular NOEs, together with increases in the heteronuclear steady-state {^1^H}-^15^N NOE values on complex formation, indicated the involvement of the C-terminal region in RNA binding. On the basis of the two complex structures, we built a structural model of consecutive RBDs with r(UAGGUAG) containing both minimal recognition sequences, which resulted in no steric hindrance. The model suggests recognition of variable lengths (*n*) of the linker up to *n* = 50 may be possible.

## 1. Introduction

Musashi (Msi) family proteins, which are highly conserved in vertebrates and invertebrates, are RNA-binding proteins that regulate the translation of target mRNAs involved in cell fate decisions. In mammals, there are two Msi paralogs, Musashi-1 (Msi1) and Musashi-2 (Msi2). They are expressed in the stem cell compartments of a variety of tissues including the hair follicles [1], germ cells [2], and neural epithelium [3], but are reportedly absent in differentiated tissues. Msi1 was found previously to play a key role in stem cell self-renewal through the down-regulation of numb mRNA, which encodes an inhibitor of Notch signaling, Numb; the reduction of Numb triggers an increase in Notch signaling [4]. Recently, Msi1 and Msi2 were found to be overexpressed in tumors of various organs and in leukemias [5,6,7,8,9,10,11,12,13,14,15,16,17]. Elucidation of the molecular mechanism by which Msi1 and Msi2 regulate the translation of targets is thereby important to understand how Msi1 and Msi2 are associated with tumorigenesis, and moreover, to understand how to therapeutically target Msi1 and Msi2.

Msi1 and Msi2 each has two RNP-type RNA-binding domains (RBDs), or RNA recognition motifs (RRMs), in the N-terminal region, followed by a putative disordered region. The RBDs confer sequence-specific nucleic acid recognition properties [4,18,19], whereas the putative disordered region primarily functions in protein-protein interactions [20,21,22]. The amino acid sequences of RBD1 and RBD2 of Msi1 exhibit 85% identity to those of Msi2, respectively [23], suggesting that the target mRNAs of Msi1 and Msi2 mostly overlap. The RNA sequences to which mouse Msi1 binds have been determined to be (G/A)U*_n_*AGU (*n* = 1–3) by using an in vitro selection method [4]. Subsequently, mouse numb mRNA was identified as an actual target of Msi1, which contains the putative Msi1 binding sequence r(UAGGUAGUAGUUUUA). Then, Msi1 was found to repress translation of cyclin-dependent kinase inhibitor p21WAF-1, which is a regulator of cell-cycle progression and arrest [24]. Later, genome-wide analysis, in which ribonucleoprotein immunoprecipitation and subsequent microarray analysis were applied to Daoy cells, revealed an enormous number of Msi1 target mRNAs. The proteins produced from those mRNAs are involved in apoptosis, cell cycle regulation, cell differentiation, cell proliferation, cell survival, and DNA repair [25].

Msi1, which binds to the 3′ untranslated region (UTR) of the target mRNA, has been proposed to compete with a translation initiation factor, eIF4G, for binding to poly(A)-binding protein (PABP) through which the assembly of the 80S ribosome complex is blocked, and thereby initiation of translation is repressed [20]. Consistent with this model, ribosome profiling (Ribo-seq) revealed that Msi1 down-regulates the translation of target mRNAs without reducing the mRNA level [26].

RBD is a single-stranded polynucleotide-binding domain that adopts a β1α1β2β3α2β4 topology, i.e., a four-stranded anti-parallel β-sheet packed against two α-helices [27]. A single RBD binds to RNA of variable length, ranging from a minimum of two to a maximum of eight nucleotides [27]. RBD has two consensus sequence stretches, RNP1 and RNP2, in the β3 and β1 strands, respectively. The canonical RBD contains three aromatic amino acid residues in RNP1 and RNP2 that are exposed on the surface of the β-sheet, which play a critical role in nucleic acid recognition. The amino acid sequences of Msi1 RBD1 (and RBD2) of mouse and human homologs exhibit 100% (and 98.9%) identity, and therefore they are expected to share structural and functional properties.

So far, we have determined the solution structures of RBD1(residues 20–103) and RBD2(109–191) of mouse Msi1, and reported that each RBD has the typical β1α1β2β3α2β4 topology, and that each contains three solvent-exposed phenylalanines in RNP1 and RNP2 [28,29]. Furthermore, we performed NMR-based binding studies on two RBDs (RBD1 and RBD2) of mouse Msi1 [18]. NMR titration experiments on Msi1 RBD1-2(20–191), an Msi1 construct containing both RBDs, involving r(UAGGUAGUAGUUUUA) indicated the multiple registration of RBD1-2 onto r(UAGGUAGU AGUUUUA), which hindered further analysis of the complex. Therefore, we analyzed the RNA-binding properties of Msi1 RBD1(20–103) and RBD2(109–191) individually. NMR titration experiments on Msi1 RBD1 or RBD2 with a series of RNA oligomers, r(UAGGUA), r(GGUAGU), r(UAGUAG), r(GUAGUU), r(AGUUUU), and r(GUUUUA), showed that RBD1 and RBD2 of Msi1 bind to r(GUAG)- and r(UAG)-containing RNA oligomers with high affinity, respectively. We then determined the solution structure of the Msi1 RBD1(20–103):r(GUAGU) complex and revealed the mechanism by which the RBD1 specifically recognizes r(GUAG). However, although the solution structure of Msi1 RBD2(109–191) in the free form was previously determined by means of homonuclear NMR spectroscopy [28], the structure of Msi1 RBD2(109–191) in complex with RNA has not been determined yet.

**Figure 1:**
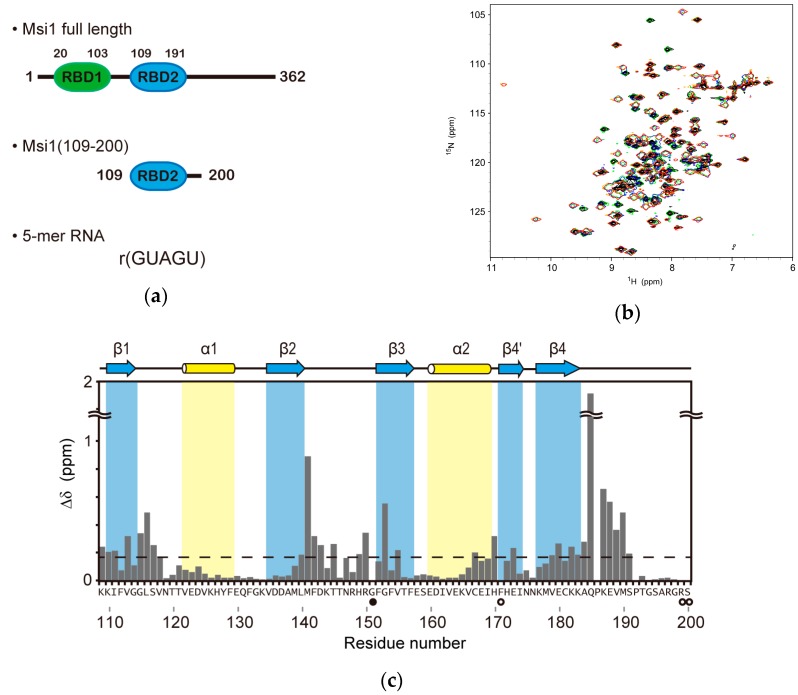
Interaction of r(GUAGU) with Msi1(109–200). (**a**) Domain organization of Msi1 (top), the protein construct used in this study (middle), and 5-mer RNA (bottom) originating from *numb* mRNA are shown; (**b**) Overlay of the ^15^N-^1^H HSQC spectra of Msi1(109–200) (80 μM) in the absence (black) and presence of r(GUAGU) with Msi1 RBD2:r(GUAGU) ratios of 1:0.25 (green), 1:0.50 (blue), 1:1.00 (yellow), and 1:1.30 (red); (**c**) The chemical shift perturbation, Δδ, was calculated using Δδ = [(ΔδH)^2^ + (ΔδN/6.5)^2^]^1/2^, where ΔδH and ΔδN are the chemical shift differences between free and complex forms for H^N^ and ^15^N, respectively. The dashed line indicates the mean value (0.167 ppm). The secondary structure elements of Msi1(109–200) are shown at the top as blue arrows (strands) and yellow cylinders (helices). The amino acid residues that were not assigned (open circles) or whose ^1^H-^15^N resonance vanished upon addition of r(GUAGU) (closed circles) are indicated beneath the amino acid sequence at the bottom.

Here, we report the three-dimensional (3D) solution structure of mouse Msi1 RBD2(109–191) (Figure 1a) in complex with r(GUAGU) for the first time; we used the Msi1 construct of the residues 109–200, Msi1(109–200), which contains RBD2(109–191). The chemical shift perturbation (CSP) of Msi1(109–200) upon r(GUAGU)-binding, and the steady-state {^1^H}-^15^N Nuclear Overhauser effects on Msi1(109–200) in the free and r(GUAGU)-bound forms were also utilized for better understanding of the interaction. Additionally, structural modeling was undertaken to integrate the knowledge obtained previously on RBD1(20–103):r(GUAGU) binding and currently on Msi1 RBD2(109–191):r(GUAGU) binding to establish the binding mode and molecular basis for the complex between consecutive RBDs of Msi1 and the target RNA sequences, r(UAGN*_n_*GUAG) (N = A/G/U/C and *n* = 0–50 nt), which are found in actual biological systems.

## 2. Results

### 2.1. Structure Determination of Msi1 RBD2(109–191) in the Free Form

The region of the residues 109–200 of Msi1, Msi1(109–200), containing RBD2(109–191) was expressed and purified to homogeneity. Resonance assignment of the uniformly ^13^C-, ^15^N-labeled Msi1(109–200) in its free form was recently accomplished [30]. Firstly, in this study, we refined the 3D solution structure of Msi1 RBD2(109–191) in the free form. A total 1116 NOE distance restraints were derived from 3D ^15^N-edited [^1^H, ^1^H]-NOESY-HSQC and ^13^C-edited [^1^H, ^1^H]-NOESY-HSQC spectra, and used in the structure calculation, together with 51 dihedral angle restraints (Table 1). The 40 structures obtained on CYANA calculation were further refined with the AMBER program, which resulted in 20 energy-refined conformers that represent the solution structure of Msi1 RBD2(109–191) (Figure S1a). Residues Lys110–Ala184 form the canonical RRM fold, i.e., a four-stranded anti-parallel β-sheet and two α-helices with a βαββαβ topology (β1: Lys110–Gly114, α1: Val122–Phe129, β2: Val135–Leu140, β3: Phe152–Phe157, α2: Glu160–Ile169, and β4: Lys177–Lys183 with an additional short β-strand: Phe171-Ile174) (Figure S1b). The C-terminal region, Gln185–Ser200, was unstructured in the free form.

### 2.2. NMR Titration Experiments on Msi1(109–200) with r(GUAGU)

Msi1 RBD2(109–191) reportedly recognizes r(UAG) [18]. We also reported the one to one stoichiometry of the RBD2(109–191):r(GUAGU) complex. Firstly, we performed NMR titration experiments on ^13^C, ^15^N-labeled Msi1(109–200) with unlabeled r(GUAGU) to investigate the RNA-binding sites of Msi1(109–200). Although the ^15^N-^1^H HSQC spectrum of Msi1(109–200) by itself contained only one set of signals, many new signals appeared during the r(GUAGU) titration (Figure 1b). This suggests that the Msi1(109–200) and r(GUAGU) binding is in the slow exchange regime on the NMR timescale. Eventually, with the molar ratio of Msi1(109–200):r(GUAGU) = 1:1, free protein signals disappeared. Further addition of r(GUAGU), resulting in excess RNA, led to no further change of the ^15^N-^1^H HSQC spectrum, confirming the 1:1 stoichiometry of the complex. These results indicate that the complex formed by Msi1(109–200) and r(GUAGU) is suitable for structural study to obtain detailed insights into the nature of the RNA binding by Msi1(109–200).

The chemical shift changes in the backbone amide nitrogens and protons between the free and bound forms of Msi1(109–200) were plotted against the residue number (Figure 1c). The Msi1(109–200) residues that showed large chemical shift perturbation were located not only on the β-sheet but also in the C-terminal region (Gln185-Ser191) of RBD2(109–191) (Figure 1c). It is noteworthy that Gln185 exhibited the largest chemical shift perturbation, and the following four residues, Lys187, Glu188, Val189, and Met190, also showed relatively large chemical shift perturbations, suggesting the presence of direct interactions between these residues and r(GUAGU), and/or indirect structural changes in the C-terminal region (Gln185-Ser191). Additionally, since the residues 193–198 showed no chemical shift perturbation, we suppose that the additional C-terminal residues are not involved in RNA-binding (192 is proline, while 199 and 200 were not assigned).

### 2.3. Structure Determination of the Msi1 RBD2(109–191):r(GUAGU) Complex

Next, we investigated the solution structure using Msi1(109–200) in a complex with r(GUAGU). Hereafter, the residues of the RNA are numbered r(G1-U2-A3-G4-U5). A total 1351 ^1^H-^1^H distance restraints, including 26 intermolecular ones and one interresidual one for RNA (Table 1), were derived from 3D ^15^N-edited NOESY-HSQC, ^13^C-edited NOESY-HSQC, two-dimensional (2D) [F1, F2] ^13^C-, ^15^N-filtered NOESY, 2D [F2] ^13^C, ^15^N-filtered NOESY, and 3D [F1] ^13^C-, ^15^N-filtered [F3] ^13^C-edited NOESY–HSQC spectra based on the resonance assignments of the uniformly ^13^C, ^15^N-labeled Msi1(109–200) in its bound form [30]. In addition to the obtained distance restraints, 19 dihedral angle restraints were used in the complex structure calculation (Table 1). The structure was determined through the combination of the CYANA 2.1 and AMBER12 programs.

**Figure 2:**
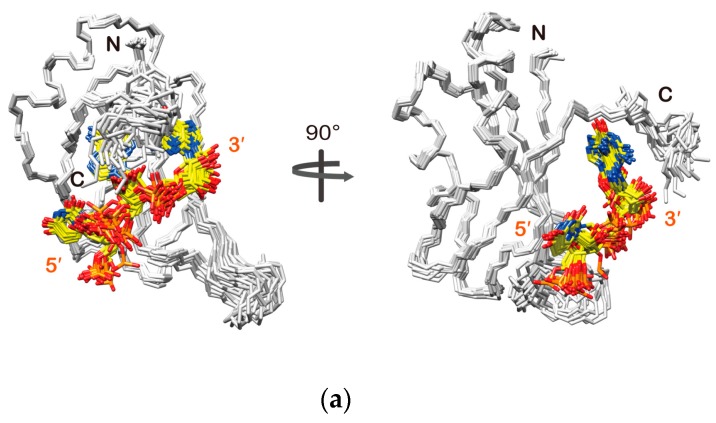

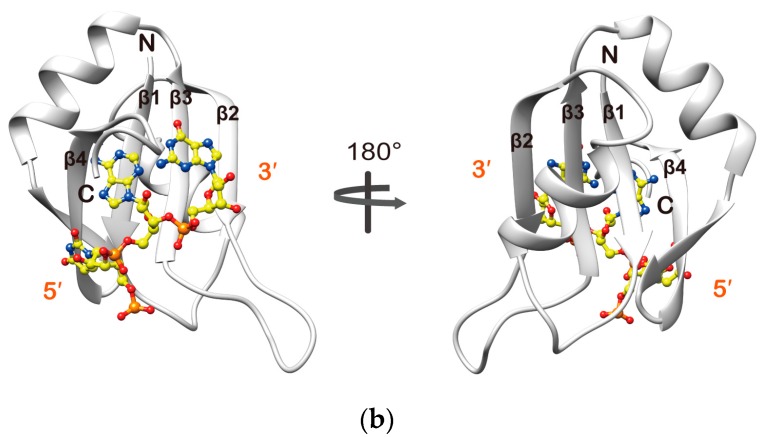
Solution structure of the Msi1 RBD2(109–191):r(GUAGU) complex. (**a**) Backbone traces of the 20 conformers of the complex are superimposed. Only the r(UAG) portion of r(GUAGU) is shown for RNA. The protein backbone (residues 109–190) is colored white. RNA is shown as a stick model: carbon (yellow), nitrogen (blue), oxygen (red), and phosphorus (orange); (**b**) Ribbon representation of the lowest energy conformer of the complex. RNA is shown as a ball-and-stick model: color-coded as in (**a**).

The region comprising residues Lys110-Ala184 in the structure obtained for the Msi1(109–200):r(GUAGU) complex takes the same RRM fold as in the structure of the RNA-free form (Figure 2). r(GUAGU) is positioned over the β-sheet surface, where the 5′ end is located on the β4-β1 region and the 3′ end on the β3-β2 region, which is a typical arrangement used by RBDs having a canonical RRM fold. The C-terminal region comprising residues Glu185-Met190 of RBD2(109–191) in the complex took a certain conformation, resembling a helix. Indeed, for residues Gln185–Met190, we observed medium-range (1 < |i − j| < 5) interresidual NOEs. This C-terminal region (185–190) did not form any structure in the free form as no medium-range (1 < |i − j| < 5) interresidual NOE was observed. However, in the presence of r(GUAGU), this C-terminal region crosses over the β-sheet surface and forms a wall on the north side of the β-sheet, as can be seen in Figure 2. Importantly, the C-terminal region (185–190) of RBD2(109–191) turned out to directly interact with the third adenine and fourth guanine (Figure 2a, right panel). These structural features of the C-terminal region (185–190) of RBD2(109–191) and its involvement in RNA-binding explain the observation of the large chemical shift perturbation, described in the previous section. Thus, the obtained structure revealed that Msi1 RBD2(109–191) interacts with r(GUAGU) on its β-sheet surface, and importantly the C-terminal region (185–190) of RBD2(109–191) is also involved in the recognition of RNA molecule.

### 2.4. Structure Description of the Base-Specific RNA Recognition by Msi1 RBD2(109–191)

**Figure 3:**
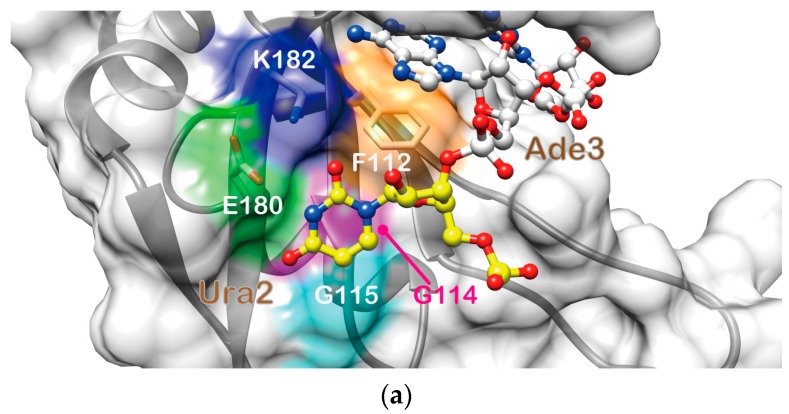

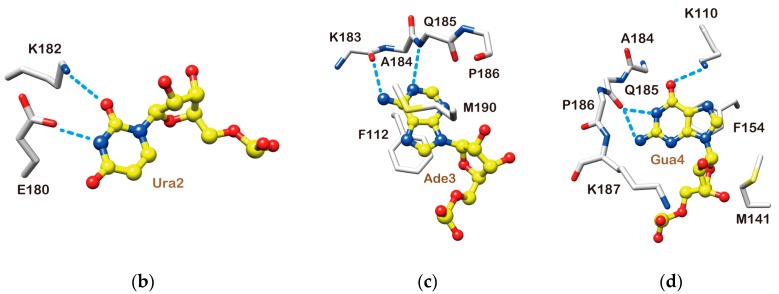
r(UAG) recognition by Msi1 RBD2(109–191). (**a**) The Ura2 base fits in a shallow basin. Msi1 RBD2(109–190) is depicted as a ribbon model with a semi-transparent molecular surface. F112 (orange), E180 (green), and K182 (blue), whose side chains are shown (oxygen and nitrogen atoms in red and blue, respectively), form a rim, while G114 (magenta) and G115 (cyan) form the bottom of the basin. RNA is shown as a ball-and-stick model: carbon is color-coded yellow (Ura2) and white (Ade3), while nitrogen, oxygen, and phosphorus are color-coded blue, red, and yellow, respectively; (**b**) Recognition of Ura2. Intermolecular hydrogen bonds, Ura2 N3-E180 Oɛ and Ura2 O2-K182 Hζ, are formed; (**c**) Recognition of Ade3. The Ade3 base stacks onto the F112 aromatic ring, and the M190 side chain likely makes a van der Waals contact with Ade3, such that the adenine is sandwiched between F112 and M190. Hydrogen bonds, Ade3 H6-K183 O and Ade3 N1-Q185 HN, are also formed. Sδ of M190 is colored yellow; (**d**) Recognition of Gua4. The Gua4 base stacks onto the F154 aromatic ring, and hydrogen bonds, Gua4 N1-Q185 O, Gua4 H2-Q185 O, and Gua4 O6-K110 Hζ, are also formed. The M141 side chain makes a van der Waals contact with the ribose of Gua4. A salt bridge between K187 Hζ and the 5′ phosphate group of Gua4 may also be formed. Sδ of M141 is colored yellow. RNA is shown as a ball-and-stick model: carbon (yellow), nitrogen (blue), oxygen (red), and phosphorus (yellow). Hydrogen bonds are indicated by cyan dotted lines.

The structure of the complex suggested that Ura2 is recognized through two hydrogen bonds: one between O2 of the Ura2 base and K182 Hζ, and the other between the imino proton of the Ura2 base and E180 Oε (Figure 3b). Additionally, the Ura2 base was found to well fit in a shallow basin on the β-sheet surface composed of F112, E180, and K182, which together form a rim, and G114 and G115, which form the bottom (Figure 3a). The NOEs that revealed the rim (F112, E180, and K182) formation were observed in the 3D ^13^C-edited [^1^H, ^1^H]-NOESY-HSQC (τ_m_ = 80 ms) spectrum (Figure S2). This interaction resulted in large chemical shift perturbation of the G115 HN resonance (Figure 1c).

The base of Ade3 was found to be likely sandwiched between the aromatic ring of F112 and the side chain of M190 (Figure 3c). This structural feature is supported by three intermolecular NOEs: F112 Hε-Ura2 H1′, M190 Hγ-Gua4 H2 and M190 Hε-Gua4 H2 (Figure S3). In addition, two intermolecular hydrogen bonds were suggested to be present: one between N1 of the Ade3 base and HN of Q185, and the other between Ade3 H6 and K183 CO (Figure 3c). Formation of the former hydrogen bond is supported by the largest chemical shift perturbation being observed for the HN resonance of Q185 (Figure 1c).

The base of Gua4 was stacked onto the aromatic ring of F154, which is one of the two conserved phenylalanines in RNP1 (Figure 3d). The NOE between H8 and H1′ of Gua4 exhibited the strongest intensity of all the observed H6/H8-H1′ NOEs, whereas the intensities of the other intra-residue NOEs of Gua4 (those between H8 and either H2′, H3′, or H5′/H5″) were either very weak or not observed. These NOE patterns indicate that Gua4 takes the syn conformation around the glycosidic bond. The observation of intermolecular NOEs between Gua4 H8/H1′ and M141 Hε supports the van der Waals contact between the M141 side chain and the ribose of Gua4. The obtained structure suggested the presence of hydrogen bonding interactions between K110 Hζ and Gua4 N7, and between Q185 CO and Gua4 H1/H2. The former hydrogen bonding is supported by the observation of a NOE between K110 Hε and F154 Hδ, which is located beneath Gua4 (Figure 3d). Additionally, a salt bridge between K187 Hζ and the 5′ phosphate group of Gua4 may also be formed, which is supported by the observation of a NOE between K187 Hα and Gua4 H2, and also by the chemical shift perturbation of the HN resonance of K187 (Figure 1c).

### 2.5. Heteronuclear Steady-State {^1^H}-^15^N Nuclear Overhauser Effect Measurement

The NMR titration experiments suggested that the C-terminal region (185–190) of RBD2(109–191) is involved in the binding with r(GUAGU) and/or undergoes a conformational change upon RNA binding. Additionally, the observation of intra- and intermolecular NOEs for the residues of the C-terminal region (185–190) of RBD2(109–191), and the obtained structure of the complex indicated that the C-terminal region directly interacts with the RNA, and takes a certain structure.

**Figure 4:**
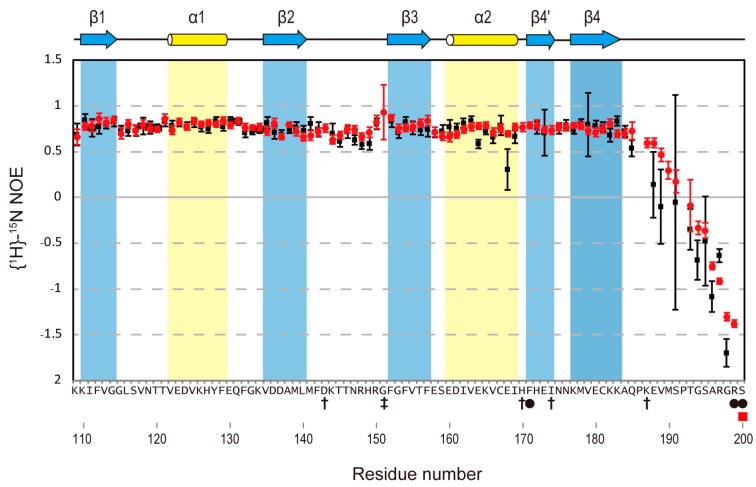
Comparison of the heteronuclear steady-state {^1^H}-^15^N nuclear Overhauser effect (NOE) values for Msi1(109–200) in its free and bound forms. The heteronuclear steady-state {^1^H}-^15^N NOE values are shown for Msi1(109–200) in the free form (black squares) and in the complex form with r(GUAGU) (red circles). The secondary structure elements (helices: yellow cylinders; β-strands: blue arrows) of Msi1(109–200) are shown at the top. The amino acid residues whose ^1^H-^15^N resonances were not assigned are indicated by black circles (free form) and a red square (complex form) beneath the amino acid sequence at the bottom. Daggers indicate the residues with overlapped ^1^H-^15^N resonances, while double-daggers indicate the residues whose ^1^H-^15^N resonance was missing in the free form.

In the absence of r(GUAGU), the {^1^H}-^15^N NOE values of the Msi1(109–200) core structure region, Lys110-Ala184, excluding the loop connecting β2 and β3, were above 0.7, indicating that the region is well-ordered. However, the {^1^H}-^15^N NOE values of the C-terminal region, Gln185-Ser200, were definitely lower than those of the core structure region, and residues Val189-Ser200 had even negative values, indicating that the C-terminal region (185–200) of Msi1(109–200) conformationally fluctuates in the free form. In the presence of r(GUAGU), the {^1^H}-^15^N NOE values of the core structure region (110–184) were basically the same as those in the free form. Interestingly, the {^1^H}-^15^N NOE values of residues Gln185-Met190 of Msi1(109–200) in the r(GUAUG)-bound form were rather larger than those in the r(GUAGU)-free form (Figure 4), although clearly less than well-ordered regions of the protein. These observations indicate that the conformational fluctuations of residues Gln185-Met190 are moderately suppressed upon complex-formation due to the direct interaction of this region with RNA, which is supported by the observation of the intermolecular NOEs between the residues Gln185-Met190 and RNA. These results are consistent with the obtained structure in which the C-terminal region is involved in the interaction with RNA.

## 3. Discussion

Among the residues in the β-sheet of RBD1(20–103) that are involved in RNA-binding, K21, F23, R61, F63, F65, D91, and K93 are conserved in RBD2(109–191), being K110, F112, R150, F152, F154, E180 and K182, respectively. D91 Oδ of RBD1 and E180 Oε of RBD2 each forms a hydrogen bond with the imino proton of Ura2, while K93 Hζ (RBD1) and K182 Hζ (RBD2) each forms another hydrogen bond with O2 of Ura2. The phenylalanines in RNP2 (on β1): F23 (RBD1) and F112 (RBD2), and those in RNP1 (on β3): F65 (RBD1) and F154 (RBD2), are used for aromatic-aromatic stacking interactions with Ade3 and Gua4, respectively. The lysines on β1: K21 Hζ (RBD1) and K110 Hζ (RBD2), approach the Gua4 base from the O6 side and form salt bridges, which seem to play a role by supporting the guanine–phenylalanine aromatic stacking. These amino acid residues in the β-sheet of RBD1 and RBD2 play key roles in the same manner to recognize the central trinucleotide, r(UAG).

In Msi1 RBD1, W29 in the β1-α1 loop recognizes Gua1 by means of an aromatic-aromatic stacking interaction [18]. Additionally, Gua1 O6 and K88 Hζ of Msi1 RBD1 form a hydrogen bond. In Msi1 RBD2, the residue corresponding to W29 in Msi1 RBD1 is V118, which cannot undergo aromatic-aromatic stacking interaction since it just has methyl groups. Indeed, the chemical shift changes of V118 and K177 (the residue corresponding to K88 of RBD1) were smaller than those of the residues involved in RNA binding (Figure 1c). Thus, the lack of interaction was the reason why Msi1 RBD2 cannot recognize Gua1.

As mentioned above, the amino acid sequences of RBD1 and RBD2 of Msi1 exhibited 85% identity to those of Msi2, respectively [23], and we found that the amino acid residues involved in the r(GUAG)- and r(UAG)-binding in Msi1 are completely conserved. Thus, Msi2 is also expected to bind specifically to r(GUAG) and r(UAG) using its RBD1 and RBD2, respectively.

**Figure 5:**
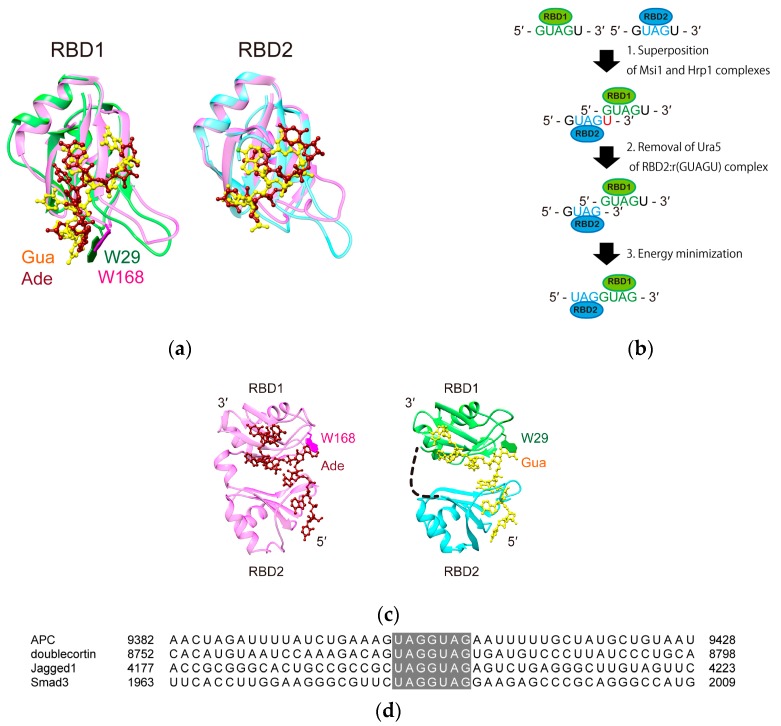
A model structure of Msi1 RBD1-2 bound to the minimal target sequence, r(UAGGUAG). (**a**) Consecutive RBDs, RBD1-2, of Hrp1 (Lys156-His322), which are connected by a short linker (Ile234–Gly243), reportedly binds to r(UAUAUAUA). The structures of the Msi1 RBD1:r(GUAG) complex (green and yellow) and Hrp1 RBD1:r(AUAU) complex (pink and red) (left), and those of the Msi1 RBD2:r(UAG) complex (cyan and yellow) and Hrp1 RBD2:r(UAU) complex (pink and red) (right) are superimposed. Note that in both pairs, the binding regions are superimposed very well. One of the characteristic interactions between Msi1 RBD1 and r(GUAG), stacking of the aromatic rings of W29 and Gua1, was similarly found in the Hrp1 RBD1:RNA complex (W168 and Ade4) (labeled in the Figure); (**b**) A schematic illustration of the model building steps; (**c**) On the basis of the similarities found in (**a**), we used the solution structure of the Hrp1:r(UAUAUAUA) complex (PDB ID: 2CJK) (left) as a template and built a model structure containing the both RBDs of both Msi1 and r(UAGGUAG) (right). The W29 in Msi1 and W168 in Hrp1 are shown as green and magenta sticks, and labelled, respectively; (**d**) Alignment of the 3′-UTR regions of the Msi family target mRNAs, containing the r(UAGGUAG) sequence (highlighted in grey): *H. sapiens* APC (accession code NM_001127511.2), *H. sapiens* doublecortin (NM_000555.3), *H. sapiens* Jagged 1 (NM_000214.2), and *H. sapiens* Smad3 (NM_005902.3).

Proteins with multiple RBDs can potentially use their RBDs to generate an extended RNA-binding interface for the recognition of their cognate RNA sequences that have different lengths and structures. This is also the case for Msi1, which has two RBDs that are connected by a linker of 14 amino acid residues. As mentioned above, Msi1 uses RBD1-2(20–191) to bind to the 3′-UTRs of its target mRNAs, containing r(GUAG) and r(UAG), which are connected by variable linkers (1–50 nt long) [18]. We have been interested to further understand how Msi1 RBD1-2 recognizes target RNA sequences of variable length, but structure determination of the complex between Msi1 RBD1-2 and targets is still technically difficult. Therefore, we searched the Protein Data Bank for the structures of consecutive RBDs bound to RNA to aid the construction of a model of Msi1 RBD1-2 bound to RNA. One of the prominent characteristics of the recognition of RNA by Msi1 is the usage of a tryptophan residue in the β1–α1 loop of its RBD1, therefore we also focused on this point when searching. As a result, we found only one other protein that uses a tryptophan residue in the β1–α1 loop of RBD1 in the consecutive RBDs for RNA-binding. This protein is Hrp1, which is involved in 3′-end processing and nuclear export of mRNAs in yeast [31]. The NMR structure clearly revealed how Hrp1 RBD1-2 binds to the target RNA, r(U$\underline{\underline{\mathrm{AU}}}$AUAUA) [32], in which Hrp1 RBD1 and RBD2 bind to the single- and double-underlined sequences, respectively. The amino acid sequence of RBD1-2 of Msi1 exhibits 48.8% identity to that of Hrp1. Despite this low identity, Hrp1 has a tryptophan, W168, in the β1–α1 loop of its RBD1, which is used to interact with adenine at the fourth position of r(U$\underline{\underline{\mathrm{AU}}}$AUAUA) (Figure 5a). The position of W168 in Hrp1 is identical to that of W29 in Msi1 (Figure 5a).

We then built a structural model of the Msi1 RBD1-2:r($\underline{\underline{\mathrm{UAG}}}$GUAG) complex using the structure of the Hrp1 RBD1-2:r(U$\underline{\underline{\mathrm{AU}}}$AUAUA) complex (PDB ID: 2CJK) as a template; hereafter, the binding sequences for RBD1 and RBD2 of either Msi1 or Hrp1 target RNA are indicated by single- and double-underlines, respectively. Firstly, we overlaid the lowest energy structure of the Msi1 RBD1:r(GUAGU) complex, which we determined previously (PDB ID: 2RS2), and that of the Msi1 RBD2:r(G$\underline{\underline{\mathrm{UAG}}}$U) complex, which we determined in this study (PDB ID: 5X3Z), onto the structure of the Hrp1 RBD1-2:r(U$\underline{\underline{\mathrm{AU}}}$AUAUA) complex. As shown in Figure 5a, the Msi1 RBD1:r(GUAGU) and Msi1 RBD2:r(G$\underline{\underline{\mathrm{UAG}}}$U) complexes superimpose very well with the Hrp1 RBD1-r(AUAUA) and Hrp1 RBD2-r U$\underline{\underline{\mathrm{AU}}}$) structural portions, respectively. At this point, we noticed that the fifth uracil residue of the Msi1 RBD2:r(G$\underline{\underline{\mathrm{UAG}}}$U) complex and the first guanine residue of the Msi1 RBD1:r(GUAGU) complex overlap and clash with each other (Figure 5b, the second row). Since, the last uracil of the Msi1 RBD2: r(G$\underline{\underline{\mathrm{UAG}}}$U) complex is not involved in the interaction, while the first guanine of the Msi1 RBD1:r(GUAGU) complex is the binding site for W29, we eliminated the last uracil of r(G$\underline{\underline{\mathrm{UAG}}}$U), keeping the first guanine of r(GUAGU), and joined them together to obtain r(G$\underline{\underline{\mathrm{UAG}}}$GUAGU) (Figure 5b, third row). Thus, we obtained a structural coordinate containing Msi1 RBD1, RBD2, and r(G$\underline{\underline{\mathrm{UAG}}}$GUAGU). Subsequently, we carried out energy minimization using these coordinates and obtained the structural model shown at the right of Figure 5c.

We successfully built a structural model of the Msi1 RBD1-2:r($\underline{\underline{\mathrm{UAG}}}$GUAG) complex. This model is reliable in the sense that it does not involve any steric hindrance. Previously, we made a list of the target RNAs for Msi1, and showed that the linkers connecting r($\underline{\underline{\mathrm{UAG}}}$) and r(GUAG) are 1–50 nt long. In the current model, we showed that Msi1 RBD1-2 can bind to r($\underline{\underline{\mathrm{UAG}}}$GUAG), which has no linker, without steric hindrance. Actually, r($\underline{\underline{\mathrm{UAG}}}$GUAG) is the Msi1-binding sequence that was found in mouse *numb* mRNA, which is regarded as a very important target for Msi1 [4]. Recently, more target mRNAs of Msi family proteins that contain r($\underline{\underline{\mathrm{UAG}}}$GUAG) have been discovered (Figure 5d); they include the mRNAs of human APC [33], doublecortin [34], Jagged1 [26], and Smad3 [35]. Therefore, it is meaningful to present a reliable structural model of Msi1 RBD1-2 bound to the minimal target sequence, r(UAGGUAG).

Several proteins with tandem RBDs are proposed to be classified into three categories based on the mode of RNA binding. The proteins in the first category, the tandem RBDs are independent of each other in the free form, but adopt certain orientation with respect to each other upon RNA binding; those in the second category, the RBDs take a fixed orientation in the free form, and retain the orientation upon RNA binding; those in the third category, the RBDs take a closed conformation occluding the RNA-binding surface in the free form, then undergo conformational change upon target RNA binding and adopt another conformation [27]. Hrp1 belongs to the first category, to which Sxl, HuD, HuR, and PABP are also the members. Most of the proteins in this first category, the two RBDs lie side-by-side forming an extended platform upon which the RNA binds in a linear manner [27]. Since, two RBDs of Msi1 are independent of each other in the free form [18], Msi1 potentially belongs to the first category. In the current study, we found that Msi1 RBD1-2 may form a complex with r(UAGGUAG) whose structure is suggested to be similar to that of the Hrp1 RBD1-2:r(UAUAUAUA) complex. Altogether, we hypothesize Msi1 to be a member of the first category.

In conclusion, we determined the solution structure of the Msi1 RBD2:r(GUAGU) complex and showed clearly how Msi1 RBD2 recognizes r(UAG). Together with our previous study involving the structure determination of the Msi1 RBD1:r(GUAGU) complex, where the recognition of r(GUAG) by Msi1 RBD1 was shown in detail, we built a model to demonstrate how consecutive RBDs, RBD1-2, of Msi1 might bind to r(UAGGUAG). Accordingly, we showed that, in theory, Msi1 RBD1-2 can target r(UAGN*_n_*GUAG) (*n* = 0–50 nt). We also found that the amino acid residues that are involved in RNA-binding are conserved in Msi1 and its paralog, Msi2. Elevated expression of Msi1 and Msi2 has been identified in a large variety of tumors arising in the bladder, brain, breast, colon, lung, ovary, pancreas, and others [5,6,7,8,9,10,11,12]. Additionally, increased expression of Msi1 and Msi2 has been found in leukemias such as acute myelogenous leukemia and acute lymphoblastic leukemia [13,14,15,16,17]. Msi1 and Msi2 are shown to be associated with tumor initiation, progression, and drug resistance [5,6,7,8,9,10,11,12,13,14,15,16,17,25,26,36,37,38,39]. Importantly, downregulation of Msi1 and Msi2 proteins in tumors results in reduction of their tumorigenic growth [40,41,42]. Thus, Msi1 and Msi2 have been receiving increasing attention in the field of medicine. Indeed, some small molecule inhibitors of Msi1 RBD1, which disrupt the RNA-binding activity of Msi1, were reported [42,43,44]. Importantly, our chemical shift assignment data (BMRB ID: 11450) and the structural coordinate (PDB ID: 2RS2) for Msi1 RBD1 were used for the screening of small molecule inhibitors [43,44]. Therefore, it is promising that the structure of the Msi1 RBD2:r(GUAGU) complex will also be useful for the screening of small molecule inhibitors.

## 4. Materials and Methods

### 4.1. Protein and RNA Preparation

The RBD2+, comprising mouse Msi1 residues Lys109-Arg200, containing RBD2, was cloned into the expression vector pET15b (Novagen), as a fusion with an N-terminal hexahistidine affinity tag and thrombin cleavage site. The protein was expressed in *E. coli* BL21 (DE3) grown in 1 L of minimal media containing either 0.1% ^15^N-NH_4_Cl or 0.1% ^15^N-NH_4_Cl and 0.3% ^13^C-Glucose as sole nitrogen and carbon sources. The former and latter conditions were used to prepare ^15^N-single labeled and ^13^C/^15^N-doubly labeled proteins, respectively. After the culture reached OD_600_ = 0.8, protein expression was induced by the addition of isopropyl-l-thio-β-d-galactopyranosid (IPTG) (final 0.5 mM) at 37 °C. The cells were harvested after 4 hours, suspended in lysis buffer containing 50 mM Tris-HCl (pH 8.0), 500 mM NaCl, 1 mM DTT, and 10 mM benzamidine and sonicated. The cell lysate was centrifuged, and the collected supernatant was loaded onto a Ni^2+^ Affinity column. After washing with wash buffer containing 50 mM Tris-HCl (pH 9.0), 2.5 M NaCl, and 1 mM DTT, protein was eluted with elution buffer containing 50 mM Tris-HCl (pH 7.0), 500 mM NaCl, 1 mM DTT, and 250 mM imidazole. The eluted protein was dialyzed against 20 mM Tris-HCl (pH 7.0) containing 50 mM NaCl, and 1 mM DTT. This protein solution was loaded onto a cation exchange column (HiTrap SP HP column, GE Healthcare, Pittsburgh, PA, USA) and further purified. The fused affinity tag was cleaved by thrombin (GE Healthcare) and removed using Ni^2+^ Affinity resin. The obtained protein contains four extra amino acid residues, Gly-Ser-His-Met, at the N-terminus. The purified protein was concentrated to 0.25–0.35 mM RBD2+ and dialyzed against 20 mM MES (pH 6.0), 100 mM NaCl, and 1 mM DTT. The final concentration of 5% D_2_O was added to make an NMR sample. The unlabeled 5-mer RNA r(GUAGU) was purchased from Sigma-Aldrich Japan. The final concentration of both ^15^N/^13^C -labeled RBD2+ and unlabeled 5-mer RNA was adjusted to be 0.3 mM.

### 4.2. NMR Spectroscopy

All NMR spectra were acquired on an AVANCE 600, DRX 600 or AVANCE III HD 950 MHz spectrometer (Bruker, Billerica, MA, USA) each equipped with a cryogenic probe, at 298 K. Resonance assignments for the uniformly ^13^C-, ^15^N-labeled Msi1(109–200) in its free and r(GUAGU)-bound forms were performed previously [30]. The resonance assignment for the r(GUAGU) in the ^13^C-, ^15^N-labeled Msi1(109–200)-bound form was performed by 2D homonuclear total correlated spectroscopy (TOCSY) [mixing time (τ_m_) = 43 ms], 2D homonuclear NOESY (τ_m_ = 80 or 200 ms), and 2D [F1, F2] ^15^N-, ^13^C-filtered NOESY (τ_m_ = 200 ms) experiments. Intermolecular NOEs involving the protons of the ^13^C-, ^15^N-labeled Msi1(109–200) and r(GUAGU) were obtained by means of 2D homonuclear NOESY (τ_m_ = 80 and 200 ms), 2D [F2] ^15^N-, ^13^C-filtered NOESY (τ_m_ = 200 ms), 3D ^13^C-edited [^1^H, ^1^H]-NOESY-HSQC (τ_m_ = 80 ms), and 3D [F1] ^15^N-, ^13^C-filtered [F3] ^13^C-edited NOESY–HSQC (τ_m_ = 200 ms) spectra. All NMR data for the structure calculations were processed using the NMRpipe software [45], and were analyzed with MagRO-NMRView [46,47,48] and Sparky [49].

### 4.3. Structure Calculations

Structure calculations for the Msi1(109–200) in its free and r(GUAGU)-bound forms were performed with the program CYANA 2.1 [50,51,52]. A chemical shift assignment table, and peak lists containing the chemical shifts and peak volumes of NOEs within the protein were created with MagRO. Protein backbone φ and ψ, and side chain χ^1^ and χ^2^ torsion angle restraints were determined by chemical shift database analysis, using the program TALOS-N [53], and were validated by inspecting the pattern of intra-residual and sequential NOE intensities [54]. Intra- and intermolecular NOEs involving the protons of r(GUAGU) in the Msi1(109–200)-bound form were manually converted into distance restraints. The conformations of the sugar rings were determined based on the intensity of the cross peaks between H1′ and H2′ in the 2D TOCSY spectra (τ_m_ = 43 ms). These data were used as inputs for CYANA 2.1 calculation for the Msi1(109–200) and Msi1(109–200):r(GUAGU) complex. Starting from 200 randomized conformers, a final ensemble of 40 conformers was selected on the basis of the lowest final CYANA target function values. The selected 40 conformers were subsequently refined using the SANDER module of AMBER12 [55] with the generalized Born solvent model and ff12SB force field, and the 20 conformers that were most consistent with the experimental restraints were then used for further analyses. The quality of the structures was analyzed using the programs UCSF Chimera [56] and PROCHECK–NMR [57]. The coordinates for the ensemble of the 20 conformers of the Msi1(109–200) and Msi1(109–200): r(GUAGU) complex were deposited in the Protein Data Bank under accession codes 5X3Y and 5X3Z, respectively.

### 4.4. NMR Titration Experiments

NMR titration experiments on ^13^C-, ^15^N-labeled Msi1(109–200) with unlabeled r(GUAGU) were performed by recording 2D heteronuclear ^15^N-^1^H HSQC spectra at 298 K. Increasing amounts of the unlabeled r(GUAGU) were added to ^13^C, ^15^N-labeled Msi1(109–200) (80 uM) to obtain molar ratios of 1:0, 1:0.25, 1:0.5, 1:1, and 1:1.3.

### 4.5. Heteronuclear Steady-State {^1^H}-^15^N Nuclear Overhauser Effect (NOE) Measurements

The steady-state {^1^H}-^15^N NOE measurements were performed at 298 K, using ^13^C-, ^15^N-labeled Msi1(109–200) in its free and bound forms. A relaxation delay of 3 s and a ^1^H presaturation time of 3 s were used in the NOE experiment, and a 6 s relaxation delay was used in the reference experiment. These experiments were recorded in an interleaved manner. The steady-state {^1^H}-^15^N NOE values were calculated as the ratio between the cross-peak intensities with and without ^1^H saturation. The errors were estimated from the root mean square of the baseline noise in the two spectra [58].

### 4.6. Model Structure Generation

A structural model of the Msi1 RBD1-2:r(UAGGUAG) complex was built in three steps (Figure 5b). Firstly, the lowest energy structure of the Msi1 RBD1(20–103):r(GUAGU) complex, which we determined previously (PDB ID: 2RS2), and that of the Msi1 RBD2(109–191):r(GUAGU) complex, which we determined in this study (PDB ID: 5X3Z), were superimposed on the Hrp1 RBD1-2:r(UAUAUAUA) complex (PDB ID: 2CJK). This procedure was carried out using the Match Maker module of UCSF Chimera. Then, the fifth uracil residue of the Msi1 RBD2:r(GUAGU) complex was removed, because this uracil residue overlapped very well with the first guanine residue of the Msi1 RBD1:r(GUAGU) complex. Subsequently, we joined the PDB coordinate files of the Msi1 RBD2:r(GUAG) and Msi1 RBD1:r(GUAGU) complexes into one, in which the coordinates of r(GUAG) and r(GUAGU) were concatenated sequentially so as to make the coordinate of a r(GUAGGUAGU) stretch. At this point, the phosphate group between the fourth and fifth guanine residues of r(GUAGGUAGU) was missing. Finally, the obtained coordinate, containing Msi1 RBD2 and Msi1 RBD1 with r(GUAGGUAGU), was subjected to an energy minimization procedure using AMBER12, by which the missing phosphate was produced, and a phosphodiester bond was formed between the fourth and fifth guanine residues of r(GUAGGUAGU), automatically. The refined model structure of Msi1 RBD1 and Msi1 RBD2 bound to r(UAGGUAG) portion of r(GUAGGUAGU) was visualized using UCSF Chimera [56] and is shown in Figure 5c.

## Figures and Tables

**Table 1:** Structural statistics for Msi1(109–200) in the free and r(GUAGU)-bound forms. (Abbreviation; NA: not applicable).

	Free Msi1(109–200)(109–200)	Msi1(109–200):r(GUAGU) Complex
NMR Restraints		
Number of NOE distance restraints	1116	1351
Intraresidue	315	341
Sequential (|i − j| = 1)	300	324
Medium-range (1 < |i − j| < 5)	165	191
Long-range (|i − j| ≥ 5)	336	468
Proteins—RNA Intermolecular	NA	26
RNA intramolecule	NA	1
Hydrogen bond restraints ^a^	27	27
Dihedral angle restraints		
φ angle	32	0
χ^1^ and χ^2^ angles	19	19
Structure statistics (20 structures)		
AMBER energies (kcal/mol)		
Mean AMBER energy	−2780	−3800
Mean restraints violation energy	4.698	4.359
Ramachandran plot statistics (%)		
Residues in most favored regions	91.5	91.5
Residues in additionally allowed regions	7.0	7.0
Residues in generously allowed regions	1.3	0.2
sidues in disallowed regions	0.2	1.4
Average RMSD to mean structure (Å)		
Protein backbone	0.55 ± 0.11 ^b^	0.74 ± 0.28 ^c^ (0.35 ± 0.07 ^b^)
Protein heavy atoms	1.45 ± 0.21 ^b^	1.45 ± 0.29 ^c^ (1.13 ± 0.15 ^b^)
Protein heavy atoms and r(UAG)	NA	1.42 ± 0.28 ^c^ (1.13 ± 0.15 ^b^)

^a^ Only used in Cyana calculation; ^b^ Residues 110–141,150–185; ^c^ Residues 110–141,150–190.

## Supplementary Materials

Supplementary Materials are available online, Figure S1: The NMR solution structure of Msi1(109–200) in the free form, Figure S2: NOEs that indicate the rim (F112, E180, and K182) formation, hydrogen-bonding, and aromatic stacking interactions, Figure S3: NOEs reveal that Ade3 was sandwiched between F112 and M190.
