# Supplementary material for: Structural Insight into the Recognition of r(UAG) by Musashi-1 RBD2, and Construction of a Model of Musashi-1 RBD1-2 Bound to the Minimum Target RNA
Source: Molecules. 2017 Jul 19;22(7):1207. doi: 10.3390/molecules22071207 (PMC6152312; doi:10.3390/molecules22071207)
Supplement: Supplementary file 1 [file molecules-22-01207-s001.pdf]

Structural insight into recognition of r(UAG) by Msi1 RBD2, and construction of a model of Msi1 RBD1-2 bound to the minimum target RNA

Ryo Iwaoka[1, 2], Takashi Nagata[1, 2*], Kengo Tsuda[3], Takao Imai[4, 5], Hideyuki Okano[4], Naohiro Kobayashi[6], Masato Katahira[1, 2*]

[1]*Institute of Advanced Energy, Kyoto University, Gokasho, Uji, Kyoto 611-0011, Japan*
[2]*Graduate School of Energy Science, Kyoto University, Gokasho, Uji, Kyoto 611-0011, Japan*
[3]*RIKEN Center for Life Science Technologies, 1-7-22 Suehirocho, Tsurumi-ku, Yokohama, Kanagawa 230-0045, Japan.*
[4]*Department of Physiology, Keio University School of Medicine, 35 Shinanomachi, Shinjuku-ku, Tokyo 160-8582, Japan*
[5]*Department of Chemistry, Keio University School of Medicine, 4-1-1 Hiyoshi, Kohoku-ku, Yokohama, Kanagawa 223-8521, Japan*
[6]*Institute for Protein Research, Osaka University, 3-2 Yamadaoka, Suita, Osaka 565-0871, Japan*

Supplemental figure legends

Figure S1. The NMR solution structure of Msi1 RBD2+ in the free form. (a) Backbone traces (residues 109-185) of the 20 conformers of Msi1 RBD2+ are superimposed. (b) A ribbon representation (residues 109-185) of the lowest energy conformer of Msi1 RBD2+. The phenylalanine residues in the RNP1 and RNP2 sequences are shown as stick models in (a) and (b).

Figure S2. NOEs that indicate the rim (F112, E180, and K182) formation, hydrogen-bonding, and aromatic stacking interactions. (a) The NOEs observed in a 3D ^{13}C-edited [^{1}H, ^{1}H]-NOESY-HSQC (τ_m = 80 ms) spectrum between H170 (Hδ2) and E180 (Hβ or Hγ) are illustrated on the determined structure (orange dashed lines). The side chains are shown as stick models: carbon (cyan), nitrogen (blue), and oxygen (red). Hydrogen bonds are indicated by green dashed lines. The residues (F112 and K182) that

are involved in the rim formation are shown as a stick model: carbon (light gray), nitrogen (blue), and oxygen (red). (b) The observed cross-peaks between H170 and E180 are shown. Note that the chemical shift values of E180 Hγ1 are upfield shifted by the ring-current of the F171 aromatic ring. (c) Illustration of the NOEs between F112 (Hδ2) and K182 (Hγ) observed by a 3D ^{13}C-edited [^{1}H, ^{1}H]-NOESY-HSQC (τ_m = 80 ms) spectrum onto the determined structure (orange dashed lines). The side chains are shown as a stick model: carbon (cyan), nitrogen (blue), and oxygen (red). Hydrogen bonds are indicated by green dashed lines. The residues (H170 and E180) that are involved in the rim formation are shown as a stick model: carbon (light gray), nitrogen (blue), and oxygen (red). RNA molecules are shown as a ball-and-stick model: carbon (light gray), nitrogen (blue), oxygen (red), and phosphorus (light gray). (d) The observed cross-peaks between F112 and K182 are shown.

Figure S3. NOEs reveal that Ade3 was sandwiched between F112 and M190. The NOEs observed in a 2D [F2] ^{15}N, ^{13}C-filtered NOESY (τ_m = 200 ms). Residues involved in RNA-binding are labeled on right side of each strip plot. (a) Ura2 H1′; (b) Ade3 H2; (c) Gua4 H2.

Supplemental Figures

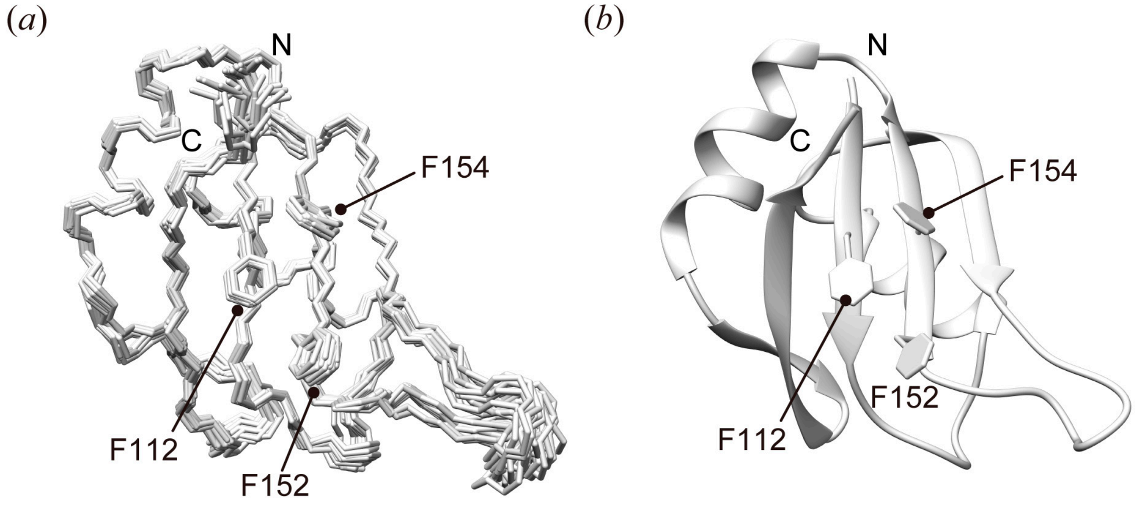

Figure S1

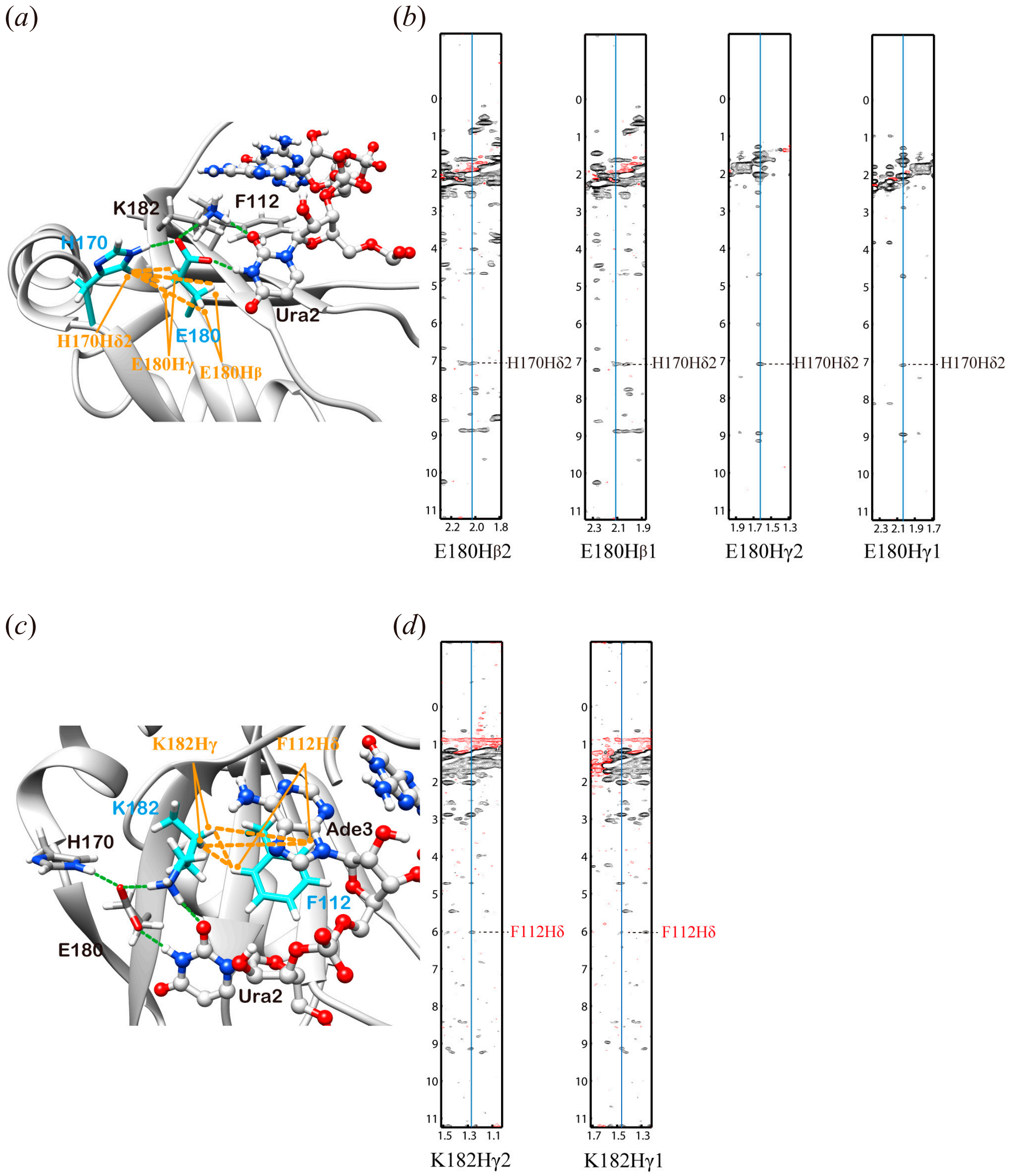
(a)
K182
F112
H170
E180
Ura2
H170Hδ2
E180Hγ
E180Hβ
(b)
H170Hδ2
E180Hβ2
E180Hβ1
E180Hγ2
E180Hγ1
(c)
K182Hγ
F112Hδ
K182
H170
Ade3
F112
E180
Ura2
(d)
F112Hδ
K182Hγ2
K182Hγ1

Figure S2

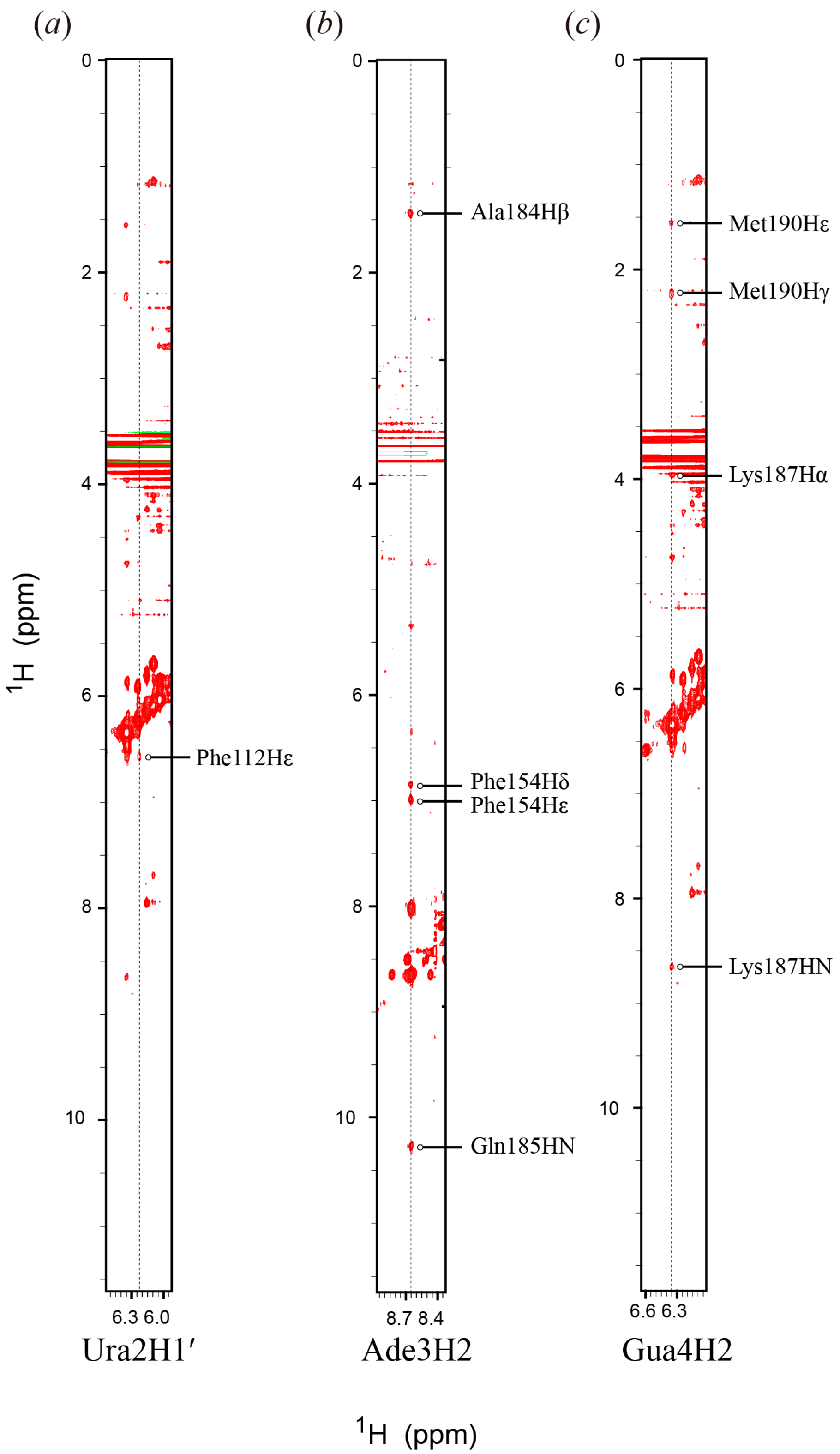
(a)
(b)
(c)
0
2
4
6
8
10
1H (ppm)
Phe112Hε
Ala184Hβ
Phe154Hδ
Phe154Hε
Gln185HN
Met190Hε
Met190Hγ
Lys187Hα
Lys187HN
6.3 6.0
8.7 8.4
6.6 6.3
Ura2H1′
Ade3H2
Gua4H2
1H (ppm)

Figure S3
